# Perceptual-cognitive training in basketball: effects on cognitive-motor performance, decision-making, and sport-specific transfer across youth players

**DOI:** 10.3389/fped.2026.1972276

**Published:** 2026-09-23

**Authors:** Mingbang Li, Yue Luo, Junlin Li, Yang Zeng

**Affiliations:** 1 College of Physical Education and Health, Sichuan Geely University of China, Chengdu, China; 2 Sichuan University of Science and Technology, Meishan, China

**Keywords:** attentional control, basketball, children, decision-making, pediatrics, perceptual-cognitive training, youth

## Abstract

**Objectives:**

To systematically identify, critically appraise, and synthesize basketball-specific perceptual-cognitive training interventions in pediatrics and youth players and examine their effects on decision-making, attentional control, cognitive-motor performance, and transfer to sport-specific or on-court performance. The review specifically focused on youth-dominant basketball samples to characterize the effects of these interventions during a developmental period relevant to adolescent sport participation.

**Methods:**

Following PRISMA 2020, PubMed, Scopus, and Web of Science Core Collection were searched. Randomized and non-randomized intervention studies involving pediatrics and youth basketball players were eligible. Two authors independently screened records, extracted data, and assessed risk of bias. Findings were synthesized narratively.

**Results:**

Fourteen eligible reports representing 11 unique participant cohorts were included from 973 records. Interventions primarily targeted attentional/gaze control, cue utilization/anticipation, decision-making/response selection, or executive-control/cognitive-motor integration. The most consistent favorable effects were observed for cognitive-motor interventions, with moderate-to-large interaction effects reported for several dribbling, sprint, change-of-direction, and selected response-control outcomes. Attentional/gaze-control evidence was limited to one small non-randomized cohort, findings for cue-utilization and anticipation training were mixed, and decision-making/response-selection evidence remained limited to two heterogeneous independent cohorts. Evidence was concentrated on trained-task and near-transfer outcomes; retention, representative game-based transfer, and competitive-match transfer remained uncommon and did not show consistent intervention-specific benefits.

**Conclusions:**

Basketball perceptual-cognitive training may improve proximal cognitive-motor and sport-performance outcomes in youth and pediatric players, particularly when executive-control demands are integrated with basketball-relevant motor tasks. However, the current evidence does not establish generalized improvements in basketball decision-making, sustained learning, or transfer to competitive performance. It also does not support conclusions regarding age- or maturation-specific responsiveness. Larger prospectively registered trials with independent samples, longer retention periods, and representative game- and match-based outcomes are needed.

**Systematic Review Registration:**

https://osf.io, Open Science Framework, unique identifier: 4zwvt.

## 1 Introduction

Basketball is a dynamic, externally paced team sport in which players must continuously perceive evolving game information, allocate attention to relevant cues, and select appropriate actions under substantial temporal pressure (1–3). Within sport science, perceptual-cognitive processes concern how performers acquire, select, and use task-relevant information to regulate action, including visual search and attentional control, cue utilization, anticipation, response selection or inhibition, and decision-making (1, 3). In basketball, these processes operate within a perception-action cycle in which information from opponents, teammates, the ball, and available space continuously constrains the actions that can be selected and executed. Accordingly, perceptual-cognitive training is distinguished here by the process intentionally targeted rather than by the technology or drill format through which training is delivered (2, 4).

Cross-sectional research on perceptual-cognitive expertise consistently shows that higher-level performers differ from lower-level performers in their extraction of advance cues and use of task-relevant visual information (1, 3). These findings identify perceptual-cognitive characteristics associated with expertise, but they do not establish that such characteristics are modifiable through training or that modifying them causes improvements in basketball performance. These questions require intervention studies in which a defined perceptual-cognitive process is deliberately trained and subsequent changes in cognitive or sport-performance outcomes are assessed. Basketball interventions have targeted attentional or gaze control, advance-cue utilization, anticipation, response selection, decision-making, and executive control under concurrent motor demands, using formats such as video or temporal-occlusion tasks, immersive simulation, gaze-oriented practice, interactive devices, and basketball-specific cognitive-motor drills (5–8). In basketball specifically, quiet-eye training has been associated with improved shooting accuracy under some conditions, and gaze-contingent perceptual training has improved decision-making skill in recreational players (7, 9, 10).

More recent basketball intervention studies have extended this work by examining cognitive-motor dual-task training, immersive simulation, and perceptual-tactical training in youth or skilled players (8, 11–13). These studies have assessed sport-specific performance, cognitive-task performance, tactical perception, and decision-making-related outcomes, although the operationalization and representativeness of these outcomes vary substantially. As illustration, assessment-focused basketball studies have also developed approaches for measuring video-based decision-making and *in-situ* gaze behavior (4, 14).

A recent systematic review and meta-analysis across team sports reported favorable effects of perceptual-cognitive training on anticipation and decision-making, while effects on real-game performance were smaller than those observed for laboratory outcomes (15). Its cross-sport, pooled perspective, however, does not resolve several questions that are specific to basketball (15–17). Consequently, the basketball evidence base remains difficult to interpret because intervention content, participant level, ecological validity, and outcome domains are heterogeneous, and the implications for decision-making, attentional control, and sport-specific transfer have not been consolidated in a single review (8, 12, 13, 16). The present review therefore distinguishes the perceptual-cognitive process intentionally targeted, the modality through which training is delivered, intervention exposure and comparator characteristics, and the level at which outcomes are assessed. In particular, it examines whether reported changes are confined to the trained task or extend to retention, near transfer, representative game-based performance, or competitive-match performance. It also maps the participant characteristics represented within the youth basketball evidence base. The purpose is therefore not to estimate a single generalized effect of perceptual-cognitive training, but to determine where basketball-specific evidence is concentrated, which intervention–outcome relationships have been examined, and where evidence for increasingly distal transfer remains limited.

Against this background, a focused systematic review on youth basketball players is needed to determine which perceptual-cognitive processes have been intentionally trained, how these interventions have been delivered, which outcomes have been assessed, and whether observed changes extend beyond trained or laboratory tasks to sport-specific and on-court performance. This question is particularly relevant to youth basketball, where perceptual, cognitive, and motor demands coexist within heterogeneous developmental and training contexts. Accordingly, the primary objective of this review was to systematically identify, critically appraise, and synthesize basketball-specific perceptual-cognitive training interventions in youth-dominant samples, with particular emphasis on decision-making, attentional control, cognitive-motor performance, and progressively more distal levels of sport-specific transfer. A secondary objective was to map the participant characteristics represented in the evidence base, specifically age, sex, basketball playing experience, and competitive level to identify gaps relevant to developmentally sensitive research. These characteristics were considered descriptively and were not interpreted as moderators of training responsiveness unless such effects were directly examined in the original studies.

## 2 Methods

This systematic review was designed for manuscript reporting in accordance with the Preferred Reporting Items for Systematic Reviews and Meta-Analyses (PRISMA 2020). Search methods and reporting elements were structured to be consistent with PRISMA-S recommendations. The review question was framed using PICOS principles, with the core elements defined as basketball players as the target population, basketball-specific perceptual-cognitive training as the intervention domain, eligible comparators ranging from usual training to active control conditions when available, and decision-making, attentional control, and transfer to sport-specific or on-court performance as the principal outcome domains. The review protocol was prospectively registered on the Open Science Framework (identification number: osf.io/4zwvt; date 13-04-2026) before the first database search was initiated on the same day. No modifications were made to the protocol.

### 2.1 Eligibility criteria

Studies were eligible when the reported mean age of the study sample was <18 years. When a mean age was not reported, studies were also eligible if the reported age range was entirely below 18 years. Studies with a mean age ≥18 years were excluded, irrespective of whether some participants were younger than 18 years. Studies were considered eligible when they evaluated basketball players of any sex or competitive level and tested a structured intervention in which modification of a perceptual or cognitive process relevant to basketball performance constituted an intentional component of the training. For this review, a perceptual-cognitive intervention was defined as one that deliberately manipulated the acquisition, selection, interpretation, or use of task-relevant information, or the selection or inhibition of an action in response to that information. Eligible target processes therefore included attentional or gaze control, visual search, cue utilization, anticipation, response selection or inhibition, decision-making, and executive control when explicitly integrated with a basketball-relevant motor task.

To qualify, the perceptual-cognitive demand had to be an identifiable and deliberately trained component of the intervention rather than an incidental consequence of performing a technical or physical basketball exercise. The intervention also had to incorporate basketball-specific information, actions, or task constraints, such as information concerning the ball, basket, teammates, opponents, spatial relations, tactical scenarios, or basketball-specific movement responses. The mode of delivery itself did not determine eligibility; video, virtual reality, augmented reality, light-based devices, gaze manipulation, or other technologies were considered delivery modalities rather than perceptual-cognitive interventions per se.

Technical skill practice, physical conditioning, or motor repetition alone was not considered perceptual-cognitive training. Likewise, generic cognitive, executive-function, reaction-time, or visual exercises were insufficient when basketball was used only as a superficial stimulus and the trained information-processing demand was not meaningfully linked to basketball action. Augmented feedback, trajectory guidance, or biomechanical instruction alone was also insufficient when it merely provided information about how to execute a movement without intentionally training information pickup, attentional regulation, anticipation, response selection, or another prespecified perceptual-cognitive process. Assessment-only exposure to perceptual-cognitive tasks was not considered an intervention.

For multicomponent or cognitive-motor interventions, eligibility required the added perceptual-cognitive component to be identifiable from the intervention description and intentionally incorporated to modify information processing or action selection while participants performed basketball-relevant activity. The addition of a secondary task or external stimulus was not, by itself, sufficient for classification as perceptual-cognitive training unless that component imposed a defined attentional, anticipatory, decisional, inhibitory, or other prespecified cognitive demand.

Eligible comparators included passive controls, usual practice, technical or physical training controls, alternative active interventions, or baseline pre-intervention measures in single-group pre-post designs. To avoid prematurely excluding a small literature, the review was intended to include randomized controlled trials, non-randomized controlled studies, quasi-experimental studies, and prospective pre-post intervention studies, provided that an explicit training intervention was delivered and outcome data were reported before and after the intervention or relative to a comparator.

Studies were required to report at least one eligible outcome in one of the prespecified domains: decision-making, attentional control, or transfer to sport-specific or on-court performance. Decision-making outcomes included, for example, response accuracy, response time, tactical choice accuracy, or other basketball-specific decision outcomes. Attentional-control outcomes included gaze behavior, quiet-eye measures, visual-search variables, selective or divided attention indices, or other measures directly related to the allocation and control of attention in basketball tasks. Transfer outcomes included basketball-specific technical, tactical, or performance indicators assessed in representative drills, pressured skill tasks, game-based tests, or match-play contexts. Basketball specificity was required for the intervention but not for every measured cognitive or neurophysiological outcome. Generic laboratory measures, such as response-inhibition tasks or event-related-potential indices, were retained only as secondary mechanistic outcomes when the intervention independently satisfied the perceptual-cognitive eligibility criteria and the study also reported an eligible basketball-relevant behavioral, attentional, or performance outcome. Changes in generic laboratory outcomes alone were not interpreted as evidence of basketball-specific transfer.

Studies were excluded if they did not satisfy the population age criterion; were observational, cross-sectional, purely descriptive, psychometric only, or assessment-only studies without an intervention; involved referees, coaches, or non-player samples; contained basketball data that could not be separated from mixed-sport samples; or were conference abstracts, editorials, commentaries, dissertations, study protocols, or review articles without original intervention data. Searches were conducted without date limits. No language filter was applied at the database-search stage.

### 2.2 Information sources

The electronic databases searched were PubMed, Scopus, and Web of Science Core Collection. The final searches were conducted on April 13, 2026. Preliminary scoping searches were undertaken in PubMed, and the terminology used in related reviews and key basketball intervention papers was examined to improve the sensitivity and face validity of the final search strings.

### 2.3 Search strategy

The search strategy was developed from main concept clusters: basketball; and perceptual-cognitive or closely related training approaches. No date restrictions were applied. Table 1 presents the specific search strategy per database.

**Table 1:** Search strategies per database.

Database	Search strategy
PubMed (NCBI/NLM)	("Basketball"[Mesh] OR basketball*[Title/Abstract]) AND ("Cognition"[Mesh] OR "Attention"[Mesh] OR "Visual Perception"[Mesh] OR "Decision Making"[Mesh] OR "Executive Function"[Mesh] OR "Psychomotor Performance"[Mesh] OR "Reaction Time"[Mesh] OR "Virtual Reality"[Mesh] OR "perceptual-cognitive"[Title/Abstract] OR "perceptual cognitive"[Title/Abstract] OR "perceptual training"[Title/Abstract] OR "cognitive training"[Title/Abstract] OR "decision-making training"[Title/Abstract] OR "decision making training"[Title/Abstract] OR "attention training"[Title/Abstract] OR "attentional training"[Title/Abstract] OR "visual training"[Title/Abstract] OR "visual attention"[Title/Abstract] OR "visual control"[Title/Abstract] OR "gaze training"[Title/Abstract] OR "quiet eye"[Title/Abstract] OR "gaze-contingent"[Title/Abstract] OR "visual search"[Title/Abstract] OR anticipat*[Title/Abstract] OR "response selection"[Title/Abstract] OR "response inhibition"[Title/Abstract] OR "reaction time"[Title/Abstract] OR "visual reaction time"[Title/Abstract] OR "video simulation"[Title/Abstract] OR "video simulations"[Title/Abstract] OR "video occlusion"[Title/Abstract] OR "temporal occlusion"[Title/Abstract] OR "spatial occlusion"[Title/Abstract] OR "immersive video"[Title/Abstract] OR "virtual reality"[Title/Abstract] OR "augmented reality"[Title/Abstract] OR stroboscop*[Title/Abstract] OR "dual-task"[Title/Abstract] OR "dual task"[Title/Abstract] OR "cognitive-motor"[Title/Abstract] OR "cognitive motor"[Title/Abstract] OR fitlight[Title/Abstract] OR "fit light"[Title/Abstract] OR "reactive agility"[Title/Abstract] OR "small-sided game"[Title/Abstract] OR "small-sided games"[Title/Abstract] OR neurotracker[Title/Abstract] OR "multiple object tracking"[Title/Abstract] OR "multiple identity tracking"[Title/Abstract] OR "perceptual tactical training"[Title/Abstract] OR "tactical perception"[Title/Abstract])
Scopus (Elsevier)	TITLE-ABS-KEY(basketball*) AND "TITLE-ABS-KEY("perceptual-cognitive" OR "perceptual cognitive" OR "perceptual training" OR "cognitive training" OR "decision-making training" OR "decision making training" OR "attention training" OR "attentional training" OR "visual training" OR "visual attention" OR "visual control" OR "gaze training" OR "quiet eye" OR "gaze-contingent" OR "visual search" OR anticipat* OR "response selection" OR "response inhibition" OR "reaction time" OR "visual reaction time" OR "video simulation" OR "video simulations" OR "video occlusion" OR "temporal occlusion" OR "spatial occlusion" OR "immersive video" OR "virtual reality" OR "augmented reality" OR stroboscop* OR "dual-task" OR "dual task" OR "cognitive-motor" OR "cognitive motor" OR fitlight OR "fit light" OR "reactive agility" OR "small-sided game" OR "small-sided games" OR neurotracker OR "multiple object tracking" OR "multiple identity tracking" OR "perceptual tactical training" OR "tactical perception")
Web of science core collection (Clarivate)	TS = (basketball*) AND TS = ("perceptual-cognitive" OR "perceptual cognitive" OR "perceptual training" OR "cognitive training" OR "decision-making training" OR "decision making training" OR "attention training" OR "attentional training" OR "visual training" OR "visual attention" OR "visual control" OR "gaze training" OR "quiet eye" OR "gaze-contingent" OR "visual search" OR anticipat* OR "response selection" OR "response inhibition" OR "reaction time" OR "visual reaction time" OR "video simulation" OR "video simulations" OR "video occlusion" OR "temporal occlusion" OR "spatial occlusion" OR "immersive video" OR "virtual reality" OR "augmented reality" OR stroboscop* OR "dual-task" OR "dual task" OR "cognitive-motor" OR "cognitive motor" OR fitlight OR "fit light" OR "reactive agility" OR "small-sided game" OR "small-sided games" OR neurotracker OR "multiple object tracking" OR "multiple identity tracking" OR "perceptual tactical training" OR "tactical perception")

### 2.4 Selection process

All records identified from the database searches were exported to EndNote and deduplicated using a combination of automated and manual procedures. Title-and-abstract screening and full-text eligibility assessment were performed independently by two authors, with disagreements resolved by consensus and, where necessary, adjudication by a third author. Reasons for exclusion at full-text review were documented. At full-text assessment, intervention eligibility was adjudicated against the criteria specified above rather than on the terminology used by the original authors. Each author independently determined (1) the perceptual-cognitive process intentionally targeted, (2) whether that process represented an active training component rather than an incidental task demand, and (3) whether the trained process was meaningfully linked to basketball-specific information or action. Studies were excluded when these requirements could not be established from the intervention description.

### 2.5 Data collection process

Data extraction was undertaken using a piloted standardized form developed for this review. Extraction was performed independently by two authors. Any disagreements were resolved through discussion. Where reports were incomplete or ambiguous, attempts were made to contact corresponding authors for clarification.

### 2.6 Data items and outcomes

For each included study, the following information was extracted: bibliographic details; country; study design; participant characteristics (sample size, age, sex, playing level, and basketball experience); intervention characteristics (primary perceptual-cognitive process targeted, additional processes targeted where applicable, delivery modality, basketball-specific information or action incorporated into the task, theoretical rationale if reported, motor/technical or physical co-components, session duration, weekly frequency, total intervention length, progression, and supervision); comparator characteristics; outcome measures; testing context; and the main quantitative findings relevant to the review question.

The primary outcome domains were decision-making and attentional control. Decision-making outcomes included basketball-specific decision accuracy, tactical-choice quality, response speed in representative decision tasks, and related measures. Attentional-control outcomes included quiet-eye duration or timing, fixation behavior, gaze allocation, visual-search behavior, selective attention, divided attention, and other direct attentional metrics relevant to basketball performance. Secondary outcomes were transfer-oriented measures, defined as sport-specific technical, tactical, or performance outcomes assessed in ecologically representative contexts, including pressured shooting tasks, dribbling or agility tests with perceptual demands, representative skill drills, and game-based or match-derived performance indicators. Additional cognitive, neurophysiological, or motor outcomes were extracted when they were presented as mechanistically relevant to the intervention effects.

### 2.7 Risk of bias assessment

Risk of bias was assessed for a prespecified result from each included report using an instrument appropriate to the study design. For reports of individually randomized parallel-group trials, the Cochrane Risk of Bias 2 tool was applied, with the effect of assignment to the intervention specified as the estimand of interest. The assessment covered bias arising from the randomization process, deviations from intended interventions, missing outcome data, measurement of the outcome, and selection of the reported result. Overall judgments were derived according to the RoB 2 decision rules rather than from a numerical domain score.

Non-randomized intervention studies with a concurrent comparison group were assessed using ROBINS-I. The target effect was defined as the intervention effect that would have been estimated in a well-conducted pragmatic randomized trial addressing the same intervention question. Before completing the ROBINS-I assessment, potentially important confounding domains were specified, including baseline perceptual-cognitive or performance level, basketball experience and competitive level, age and sex where relevant, playing position, team and coaching context, and concurrent training exposure. The seven ROBINS-I domains were then considered: confounding, selection of participants, classification of interventions, deviations from intended interventions, missing data, measurement of outcomes, and selection of the reported result.

The uncontrolled sequential intervention study was appraised using the revised Joanna Briggs Institute critical appraisal tool for quasi-experimental studies. The JBI items were considered individually, including temporal precedence of the intervention, presence of a comparison group, comparability and treatment of participants, repeated measurements, consistency and reliability of outcome assessment, completeness of follow-up, and appropriateness of the statistical analysis. Because the JBI tool does not prescribe a numerical summary score, item-level judgments were retained and no overall quality percentage or arbitrary cut-off was calculated.

Risk-of-bias assessments were specific to the results. Before appraisal, the outcome, statistical result, and assessment time point contributing to the review synthesis were identified for each report. When several eligible outcomes or analyses were available, the result most directly corresponding to the review outcome domain and the principal post-intervention assessment was selected; distinct results reported in companion publications were appraised separately when they contributed independently to the synthesis. Reports arising from the same participant cohort were explicitly linked and were not treated as independent studies when summarizing the distribution of risk-of-bias judgments or conducting cohort-level sensitivity analyses.

Assessments were performed independently by two authors, with disagreements resolved through discussion and consensus. Risk-of-bias judgments were incorporated into the interpretation of the synthesis, and sensitivity analyses restricted the evidence base by excluding non-randomized studies at serious or critical risk of bias and uncontrolled studies.

### 2.8 Effect measures

For each eligible outcome, quantitative results were extracted as reported in the original article. Preference was given to estimates that most directly represented the intervention effect. For controlled studies, group-by-time interactions, adjusted between-group contrasts, or other comparative estimates were prioritized over separate within-group changes. Where a direct comparative estimate was not reported, group-specific pre- and post-intervention data and the corresponding inferential statistics were retained and interpreted cautiously. For uncontrolled studies, within-participant change estimates were extracted but were not considered equivalent to comparative intervention effects.

Effect measures were retained in their reported form because the included studies differed substantially in design, outcome scale, and statistical model. These included mean and standard deviation values, mean or percentage changes, F, t and non-parametric test statistics, partial *η*^2^, Cohen's d, Kendall's W, correlation or regression coefficients, and associated *p* values. Sample size contributing to the analysis, assessment time point, and whether the reported estimate was adjusted were also extracted when available. Numerical 95% confidence intervals were recorded when reported by the study authors. When confidence intervals were shown only graphically, or the information required for their valid reconstruction was unavailable, numerical limits were not estimated from the figures.

No missing standardized effect size was imputed from incomplete reporting, and effect estimates were not converted across statistical metrics solely to create a common numerical scale. This avoided introducing additional assumptions into a clinically and methodologically heterogeneous evidence base. Statistical significance was not used as the sole criterion for interpreting an intervention effect. The synthesis considered the magnitude and direction of the reported estimate, study design, sample size, consistency across related outcomes, transfer level, and risk of bias. Companion publications from the same participant cohort were linked so that multiple reports were not interpreted as independent replications.

### 2.9 Synthesis methods

Interventions were coded on two separate dimensions: the perceptual-cognitive process targeted and the modality through which training was delivered. Target processes were classified as attentional/gaze control, cue utilization or anticipation, decision-making/response selection, or executive-control/cognitive-motor integration; more than one process could be coded when explicitly targeted. Delivery modalities were coded separately and included live or on-court practice, two-dimensional video or computer-based simulation, immersive or virtual-reality formats, interactive-device or light-based training, and mixed delivery. The primary synthesis was organized according to the process targeted, with delivery modality used as a secondary descriptive characteristic rather than as the definition of the intervention itself.

Outcomes were additionally classified according to their distance from the trained task. Trained-task acquisition referred to performance assessed immediately after the intervention using the same task, stimulus structure, or practiced information-action relation used during training. Retention was considered separately from transfer and was defined as performance on the same or an equivalent trained task after a delay during which the experimental training was no longer provided. Near transfer referred to performance on an untrained or modified task that retained the principal perceptual-cognitive demand of the intervention but changed the specific stimuli, response requirements, or task context. Far transfer referred to outcomes assessed in substantially more representative performance environments and was subdivided into representative game-based transfer, such as small-sided or structured game play, and competitive-match transfer derived from actual competition. Classification was performed for individual outcomes rather than entire studies because a single report could contribute outcomes at different levels. Generic cognitive and neurophysiological measures were coded separately as mechanistic outcomes and were not interpreted as evidence of sport-specific transfer.

## 3 Results

### 3.1 Study selection

Figure 1 summarizes the flow chart process. A total of 973 records were identified from database searching, comprising 156 from PubMed, 437 from Scopus, and 380 from Web of Science Core Collection. Before screening, 426 duplicates were removed. This yielded 547 unique records for title-and-abstract screening.

**Figure 1:**
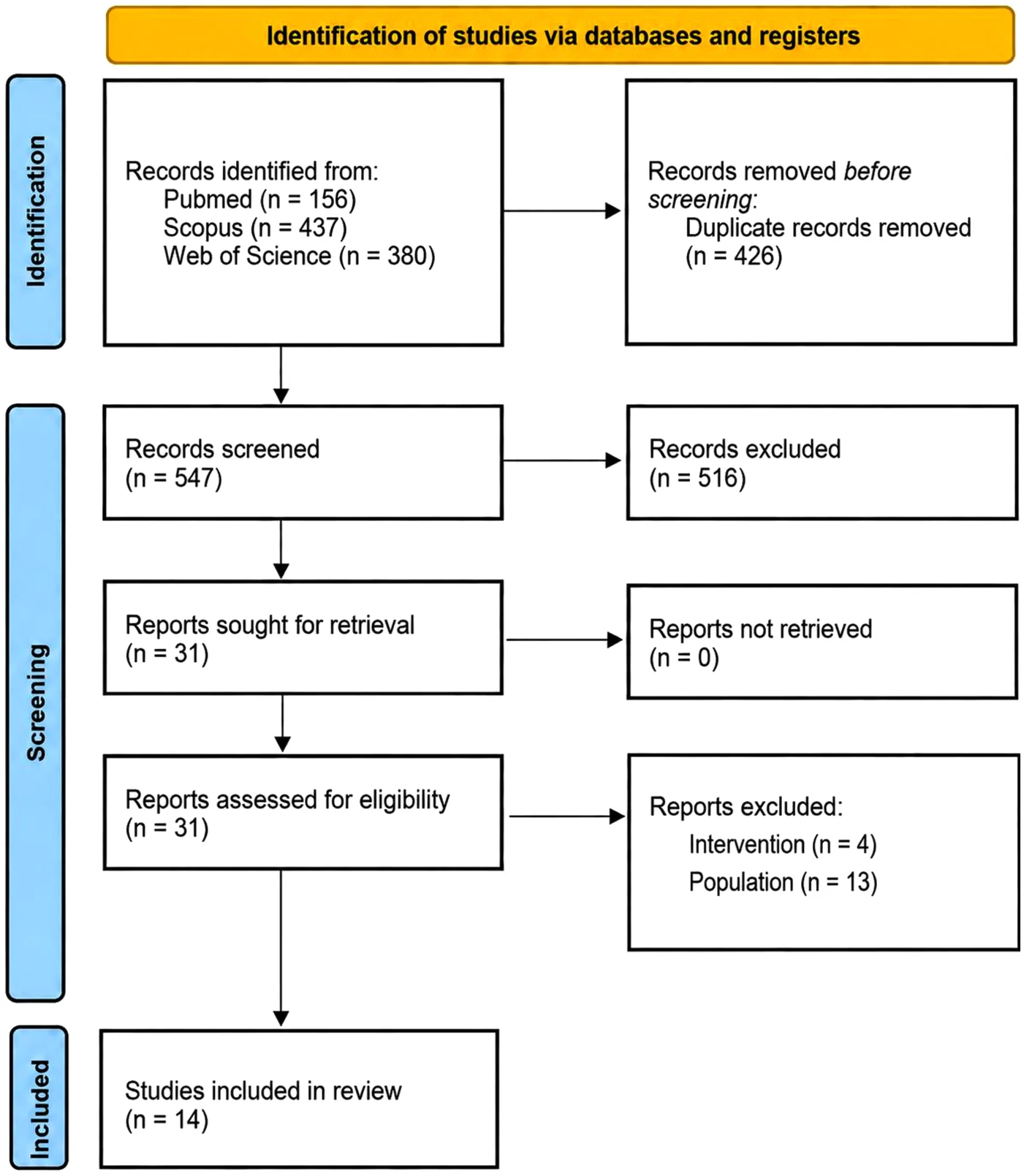
PRISMA flow diagram.

At title-and-abstract screening, 516 records were excluded and 31 reports progressed to full-text assessment. Of the 31 full-text reports assessed, the following were excluded: 11 were excluded because age was > 18 years old, 2 because the intervention was not a basketball-specific perceptual-cognitive training intervention, 2 because the intervention was not delivered in a basketball-specific perceptual-cognitive context, 1 because basketball-player data were not separable from a mixed non-player sample, and 1 because the population was not restricted to basketball players. Fourteen reports were included in the review.

The 14 included publications represented 14 eligible reports but eleven unique participant cohorts. Three pairs of companion reports were identified. The studies (13, 18) were derived from the same 24-player cohort. Similarly, two studies (8, 19) reported different analyses from the same 52-player cohort. Finally, the studies (11, 20) were based on the same 30-player cohort. All reports were retained when they contributed distinct outcomes or analyses, but companion reports were treated as a single cohort when counting independent samples and constructing the evidence gap map.

### 3.2 Characteristics of included studies

Table 2 summarizes the characteristics of the eligible reports. The reports were published between 2005 and 2025 and originated from Spain, Australia, Italy, Brazil, Netherlands and Saudi Arabia. Participants were generally adolescent basketball players competing in school, academy, club, national-development, or semi-elite contexts.

**Table 2:** Characteristics of the included studies.

Study/report	Population/design	Primary perceptual-cognitive target	Intervention dose	Comparator dose	Adherence/attrition	Follow-up	Main outcomes
Ximenes de Andrade et al. (23)	Brazil; 6 male youth players, 14–18 years. Sequential within-cohort pilot with two active phases separated by wash-out.	Executive control/cognitive-motor integration; response inhibition, anticipation and decision-related processing.	Duration: 8 weeks standard motor/basketball training + 8-week wash-out + 8 weeks cognitive-motor dual-task (CMDT) training (24-week sequence). Frequency: 2 sessions/week in each phase. Session duration: phase 1, 60 min (10 min standard motor + 50 min basketball); wash-out, 50 min basketball; CMDT phase, 60 min (10 min CMDT + 50 min basketball).	Within-participant comparison. Standard-training phase: 2 × 60 min/week; wash-out: 2 × 50 min/week. No concurrent control group.	Adherence: session-level attendance NR; all 6 participants completed the study. Attrition: none reported among the analysed sample (*n* = 6).	Four assessments: pre/post standard phase and pre/post CMDT phase. No delayed retention assessment after CMDT.	Five NBA Draft Combine dribbling tests; Go/No-Go correct responses and response time.
Diaz-Rodriguez et al. (18)	Spain; 24 male junior players, 16–17 years; experimental *n* = 16, control *n* = 8. Non-randomized controlled pre-post study.	Cue utilization/anticipation; decision-making/response selection.	Duration: 13 consecutive weeks. Frequency: 3 sessions/week (Monday, Wednesday, Friday); week 1 included pre-testing and week 13 post-testing. Session duration: approximately 30 min; 25 basketball situations with progressive exposure restriction (3.0 to 2.0 s) and feedback.	Conventional team basketball training over the same 13-week period; comparator session duration/weekly dose not quantified.	Adherence: NR. Attrition: no attrition reported; final analyses retained experimental *n* = 16 and control *n* = 8.	Post-intervention assessment in week 13; no delayed retention/follow-up reported.	Meaningful tactical perception; tactical-solution quality; game-related tactical performance measures.
Diaz-Rodriguez and Perez-Cordoba (13)	Spain; 24 male junior players, 16–17 years; experimental *n* = 16, control *n* = 8. Companion analysis of the same non-randomized cohort.	Cue utilization/anticipation; decision-making/response selection.	Duration: 13 consecutive weeks. Frequency: 3 sessions/week (Monday, Wednesday, Friday). Session duration: approximately 30 min; same perceptual tactical training with 25 basketball situations, progressive time restriction and feedback.	Conventional team basketball training over the same 13-week period; comparator session duration/weekly dose not quantified.	Adherence: NR. Attrition: no attrition reported; all 24 participants underwent pre- and post-intervention assessment.	Post-intervention assessment in week 13; no delayed retention/follow-up reported.	Tactical perception and tactical-solution quality; Raven matrices and Eysenck dimensions as predictors of response/originality.
Gorman and Farrow (22)	Country not explicitly stated; 39 skilled male players, mean age 17.84 +/− 2.11 years. Four-group non-randomized comparative study.	Cue utilization/anticipation; decision-making/response selection.	Duration: 4 weeks. Frequency: 3 sessions/week (12 sessions). Session duration: NR; explicit/implicit groups viewed 30 clips/session (360 clips total).	Placebo completed the same amount of training (12 sessions: 6 Acuvision light-board + 6 pattern-recall sessions). No-training control completed testing/transfer only.	Adherence: training-session attendance NR. Attrition: 39 at baseline; 1 placebo participant missed retention due to injury (retention *n* = 38); transfer data available for 29 because of injury or insufficient court-time.	Immediate post-test; 14-day unfilled retention test; competitive-game transfer assessment.	Video-based decision accuracy; retention; decision-making performance during competitive games.
Granda Vera et al. (24)	Spain; 22 male federation youth players, mean age 15.4 years. Three-arm non-randomized controlled study.	Cue utilization/anticipation; decision-making/response selection.	Duration: two consecutive 2-month intervention periods (approximately 4 months total). Frequency: experimental on-court groups, 4 sessions/week; computer-simulation group additionally received 2 Reflex sessions/week. Session duration: on-court sessions 1.5 h; Reflex session duration NR (10 sequences/session).	Control continued usual on-court/team training with no additional treatment; exact comparator dose NR.	Adherence: NR; analyses were based on participants who completed the study with complete datasets. Attrition: initial recruited number versus completers not separately reported.	Pre-test; retest after first 2 months; post-test after second 2 months; higher-complexity transfer test at the end. No delayed retention assessment.	Anticipation ability index and reaction time in the practiced 1-v-1 task; higher-complexity transfer test.
Hassan et al. (26)	Saudi Arabia; 20 youth players; experimental *n* = 10, control *n* = 10. Two-group controlled pre-post study; allocation method NR.	Decision-making/response selection; visually cued reaction and reactive agility integrated with dribbling.	Duration: 8 weeks. Frequency: 4 training units/week (32 units). Session duration: FITLIGHT reactive-agility component 35-45 min after a standardized 15-min warm-up.	Traditional training over the same 8-week, 4-unit/week calendar without FITLIGHT reactive-agility exercises; exact time-equivalence of the added component not separately reported.	Adherence: attendance percentage NR; researchers supervised both groups. Attrition: no losses reported; *n* = 20 included in post-test comparisons.	Post-testing performed 2–3 days after the final training date; no delayed follow-up/retention reported.	Modified agility t-test; right/left visual reaction time; modified agility t-test with basketball dribbling.
Lucia et al. (25)	Italy; 24 male semi-elite U18 players, 16.6 +/− 1.1 years; randomized trial, 12 experimental and 12 control.	Executive control/cognitive-motor integration; anticipation, inhibition, working memory and decision-making.	Duration: 5 weeks. Frequency: overall schedule 7 basketball exposures/week (1 match + 6 training days), including 2 individual sessions/week. Session duration: team training 3 h; match 2 h; individual CMDT sessions 30 min.	Same overall team/match schedule; the two weekly 30-min individual sessions used standard physical/technical motor training instead of CMDT.	Adherence: session-level attendance NR; all 24 participants completed the pre/post assessments. Attrition: none reported.	Basketball tests 2 days and cognitive/EEG tests 1 day after treatment. No retention test was conducted.	Five dribbling tests; discrimination-response accuracy/time; Bereitschaftspotential and prefrontal negativity.
Lucia et al. (19)	Italy; 52 semi-elite players (28 female, 24 male), approximately 16 years; randomized trial, 26 experimental and 26 control.	Executive control/cognitive-motor integration; decision-making/response selection.	Duration: 5 weeks. Frequency: 7 basketball exposures/week (1 match + 6 training days), including 2 individual sessions/week. Session duration: team training 3 h; match 2 h; individual CMDT sessions 30 min.	Same overall team/match exposure; two weekly 30-min individual sessions consisted of standard physical/technical motor training.	Adherence: session-level attendance NR; 52 participants were included in the reported pre/post analyses. Attrition: no participant loss reported in the analysed cohort.	Immediate post-intervention assessment; no delayed retention/follow-up reported.	Basketball dribbling; discrimination-response speed/accuracy; reactive ERP components P1, N1 and P3.
Lucia, Aydin and Di Russo (8)	Italy; same 52-player semi-elite U18 cohort (28 female, 24 male). Sex-stratified companion randomized report.	Executive control/cognitive-motor integration; anticipation, response inhibition and decision-making.	Duration: 5 weeks. Frequency: 7 basketball exposures/week (1 match + 6 training days), including 2 individual sessions/week. Session duration: team training 3 h; match 2 h; individual CMDT sessions 30 min.	Same overall team/match exposure; control completed two weekly 30-min standard individual physical/technical sessions.	Adherence: session-level attendance NR; all 52 participants completed pre/post testing. Attrition: none reported.	Basketball tests 2 days and cognitive/EEG tests 1 day after intervention; no delayed retention reported.	Dribbling; discrimination-response time/accuracy; Bereitschaftspotential and prefrontal negativity, analysed by sex.
Lucia et al. (20)	Italy; 30 elite youth players (15 female, 15 male), 15-17 years; randomized trial, 15 experimental and 15 control.	Executive control/cognitive-motor integration; decision-making/response selection and visual-search demands.	Duration: 5 weeks. Frequency: weekly programme included five 3-h basketball sessions + a weekend match; within this schedule, 2 physical sessions/week. Session duration: the two sprint/agility sessions were 30 min each.	Same weekly basketball/match exposure and the same 2 × 30-min physical circuits; control performed motor-only multicomponent training without cognitive tasks.	Adherence: session-level attendance NR; all 30 participants completed the pre/post tests. Attrition: none reported.	Physical tests 2 days and cognitive/EEG tests 1 day after intervention; no delayed retention. Authors specifically recommended future follow-up analyses.	10-m sprint; change of direction; Go/No-Go response time/accuracy; P3 ERP.
Lucia et al. (11)	Italy; same 30-player skilled cohort (15 female, 15 male), mean age 16.1 +/− 1.1 years. Companion randomized report.	Executive control/cognitive-motor integration; fast decision-making and motor preparation.	Duration: 5 weeks. Frequency: 6 basketball exposures/week (1 match + 5 team training days), including 2 physical sessions/week. Session duration: team training 3 h; match 2 h; physical CMDT sessions 30 min.	Same weekly programme; control completed the same 2 × 30-min physical circuits with the same coach but without the cognitive component.	Adherence: session-level attendance NR; all 30 participants completed the outcome testing. Attrition: none reported.	Physical tests 2 days and cognitive/EEG tests 1 day after treatment; no delayed follow-up/retention reported.	Sprint and agility; discrimination-response time/accuracy; anticipatory/preparatory ERP measures.
Oudejans et al. (27)	Netherlands; National Youth League U21 team. Experimental *n* = 6 (mean age 17 years); team comparison *n* = 4 (mean age 17.5 years). Non-randomized intervention.	Attentional/gaze control; education of attention and temporal visual-information pickup during jump shooting.	Duration: 8-week visual-control intervention. Frequency: 4 laboratory sessions across 8 weeks + 11 screen sessions organized once/twice weekly during team practice. Session duration: time NR; laboratory session included 150 test shots; screen training included 50 shots/session.	Comparison players followed normal team training and received additional coach-supervised practice/shooting drills; they did not enter the structured intervention/measurement sessions. Comparator time dose NR.	Adherence: intervention players attended 6–10 of the 11 screen sessions. Attrition: laboratory/experimental datasets were incomplete because of injuries, time constraints or technical failure; analyses used 3–5 of the 6 intervention players depending on outcome.	Competitive-game shooting was monitored before, during and after intervention; the post-intervention phase covered the final 2 months of the season. No formal delayed laboratory retention test.	Final visual-information period; laboratory full-vision shooting; competitive field-goal and three-point percentages.
Panchuk et al. (6)	Australia; elite national U19 players; 20 volunteered and 18 were analysed. Coach-constrained controlled pre-post study.	Decision-making/response selection; cue utilization/anticipation.	Duration: 3 weeks. Frequency: female training players completed 10 sessions; male training players 12 sessions. Session duration: 5 min/session, 15 immersive 360-degree clips/session, immediately before regular practice.	Both groups continued usual basketball practice; control received no additional immersive-video training and participated only in study testing/SSG assessments.	Adherence: all training players completed the scheduled 10 (female) or 12 (male) supervised sessions; session count was included as a covariate. Attrition: 20 volunteered; 1 could not participate because of injury and 1 could not complete testing because of HMD-induced motion sickness; final *n* = 18.	Post-test in the week after training, including immersive test and 1–2 SSGs. No retention test was conducted.	Immersive-video decision-making score; on-court small-sided-game performance.
Silvestri et al. (21)	Italy; 58 male U15/U17 players randomized; 49 completed/analyzed, mean age 15.0 +/− 1.5 years. Three-week massed-training trial.	Executive control/cognitive-motor integration; attentional control/peripheral awareness and decision-making.	Duration: 3 weeks. Frequency: 5 training days/week (15 intervention days), one 2-h team session/day. Session duration: 25 min/day of FITLIGHT-enriched work within the daily basketball session.	Same 3-week, 5-day/week massed basketball schedule; control completed a matched 25-min shooting-drill component instead of FITLIGHT training.	Adherence: session attendance percentage NR; 49 players completed the training protocol. Attrition: 9/58 (15.5%) were excluded during intervention because of injury, COVID-19 vaccination or illness; final *n* = 49 (24 control, 25 FITLIGHT).	Immediate post-intervention testing only; no delayed retention/follow-up reported.	Flanker/Reverse Flanker; Digit Span; Agility T-test; Yo-Yo IR1; perceived exertion and enjoyment.

Adherence refers to session attendance or completion only when explicitly reported in the article.

NR, not reported.

Study design also varied across the evidence base. Six reports, arising from four unique cohorts, described randomized parallel-group interventions; seven reports, representing six unique cohorts, used non-randomized comparative designs; and one report used an uncontrolled sequential within-participant intervention. The two Díaz-Rodríguez et al. reports (13, 18) were treated as companion publications from the same 24-player perceptual-tactical intervention. Two additional companion-report pairs were identified among the Lucia studies: reports (8, 19) arose from the same 52-player cohort, whereas reports (11, 20) arose from the same 30-player cohort. These publications were retained when they contributed distinct outcomes or analyses but were not counted as independent participant samples.

The interventions were classified according to the perceptual-cognitive process intentionally targeted rather than the technology or training format used to deliver the intervention. Across the 11 unique cohorts, one primarily targeted attentional/gaze control, three targeted cue utilization or anticipation, two targeted decision-making or response selection, and five targeted executive control or cognitive-motor integration. At report level, these categories accounted for one, four, two, and seven reports, respectively. Attentional/gaze-control training was represented by visual-control manipulation during basketball shooting; cue-utilization and anticipation interventions included temporal-occlusion, video-supported perceptual training, and perceptual-tactical practice; decision-making/response-selection interventions included immersive video and visually cued FITLIGHT reactive-agility training; and executive-control/cognitive-motor integration was principally represented by basketball-specific dual-task protocols and interactive stimulus-response training. Delivery modality was therefore treated as a descriptive feature rather than as the defining characteristic of the intervention.

Training exposure ranged from short, concentrated interventions to programmes extending across several weeks or months. Most interventions were delivered repeatedly alongside regular basketball practice, commonly using active comparison conditions such as conventional basketball training, technical practice, or motor training. Follow-up was predominantly restricted to immediate post-intervention assessment. Formal retention testing was uncommon, and direct assessment of transfer to game-based or competitive performance was considerably less frequent than laboratory, drill-based, or proximal basketball-performance testing.

Figure 2 maps the distribution of the evidence across the four perceptual-cognitive target categories and the outcome and transfer domains. To avoid inflation from multiple outcomes or companion publications, each cell represents the number of unique participant cohorts contributing at least one eligible outcome, rather than the number of reports, individual variables, statistical comparisons, or time-point-specific estimates. Thus, the 14 eligible reports were consolidated into 11 unique participant cohorts for the evidence gap map.

**Figure 2:**
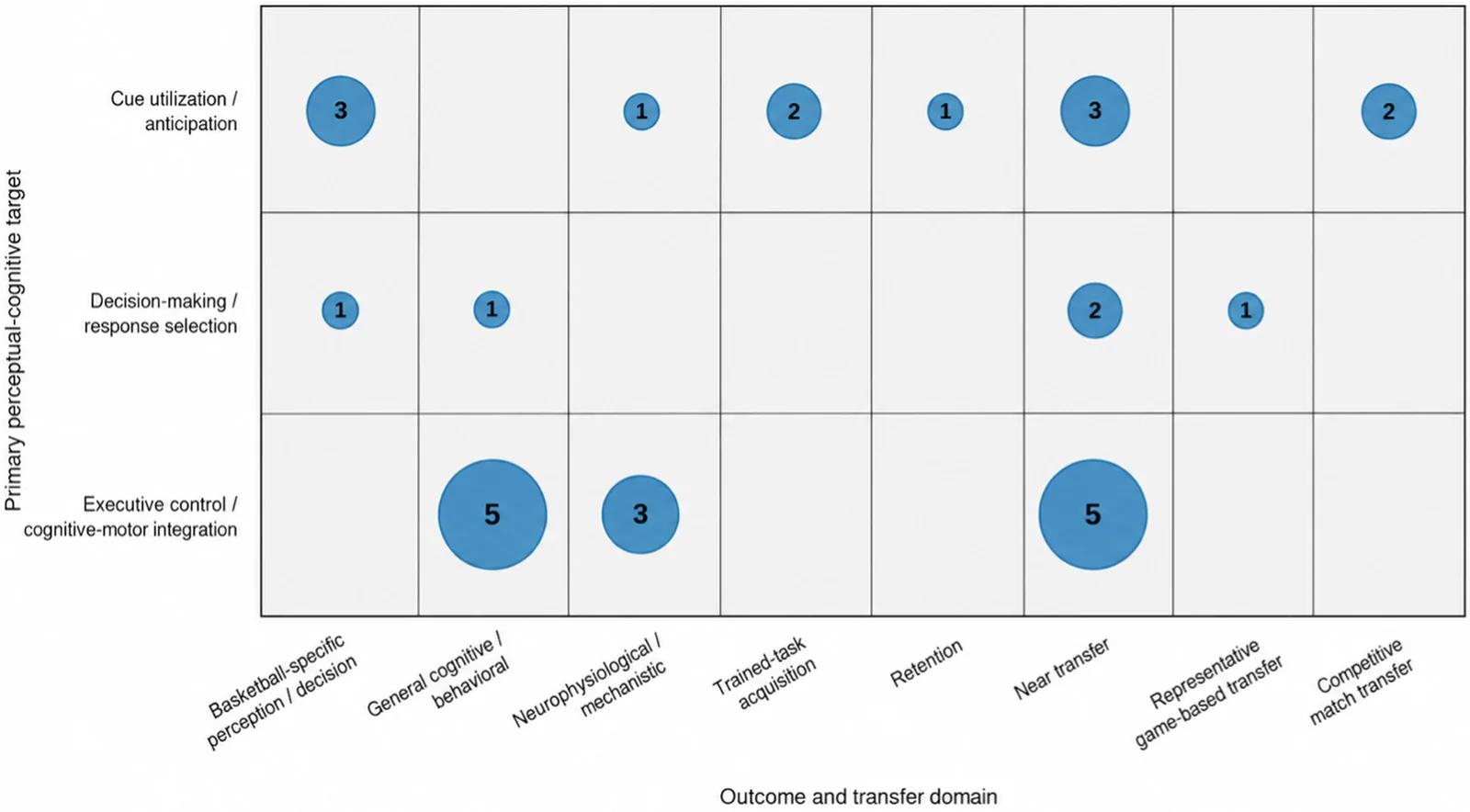
Evidence gap map of perceptual-cognitive training outcomes in youth-dominant basketball samples. The bubble area and the number within each bubble indicate the number of unique participant cohorts that contributed at least one eligible outcome to the corresponding cell.

When outcomes were organized according to transfer distance, the evidence was concentrated at the proximal end of the hierarchy. Trained-task acquisition was explicitly represented in two cohorts: Granda Vera et al. (24), through post-intervention performance on the practiced 1-v-1 anticipation task, and Oudejans et al. (27), through visual-control performance under the trained shooting conditions. Formal retention was assessed in only one cohort, Gorman and Farrow (22), using a 14-day unfilled retention interval. Near-transfer outcomes were more common and were reported across ten unique cohorts, including modified or untrained basketball-performance, perceptual, cognitive-motor, dribbling, agility, sprint, and decision tasks. Far transfer was uncommon. Panchuk et al. (6) provided the only representative game-based transfer assessment through small-sided games, whereas Gorman and Farrow (22) and Oudejans et al. (27) assessed outcomes during actual competitive matches. Thus, evidence of immediate or closely related task adaptation was substantially more common than evidence of persistence or transfer to representative competitive performance.

The evidence remained unevenly distributed. The single attentional/gaze-control cohort contributed a basketball-specific perceptual measure together with trained-task, near-transfer, and competitive-match outcomes. All three cue-utilization/anticipation cohorts contributed basketball-specific perceptual or decision outcomes; two contributed near-transfer evidence, while trained-task acquisition, retention, and competitive-match transfer were each represented by one cohort. The two decision-making/response-selection cohorts contributed complementary evidence: one assessed basketball-specific decision-making, near transfer, and representative small-sided-game transfer, whereas the other assessed general visual-response performance and near-transfer agility/dribbling outcomes. The five executive-control/cognitive-motor integration cohorts remained concentrated in general cognitive or behavioral and near-transfer outcomes, with three also contributing neurophysiological measures. None of these five cohorts assessed representative game-based or competitive-match transfer.

### 3.3 Risk of bias and critical appraisal

Six reports derived from four randomized cohorts were assessed using RoB 2 (Figure 3), seven reports representing six non-randomized comparative cohorts were assessed using ROBINS-I (Figure 4), and one uncontrolled sequential intervention study was appraised using the revised JBI quasi-experimental tool (Figure 5). Companion reports were retained when they contributed distinct results to the synthesis, but were linked to their underlying cohort and were not counted as independent studies when the overall distribution of methodological limitations was interpreted.

**Figure 3:**
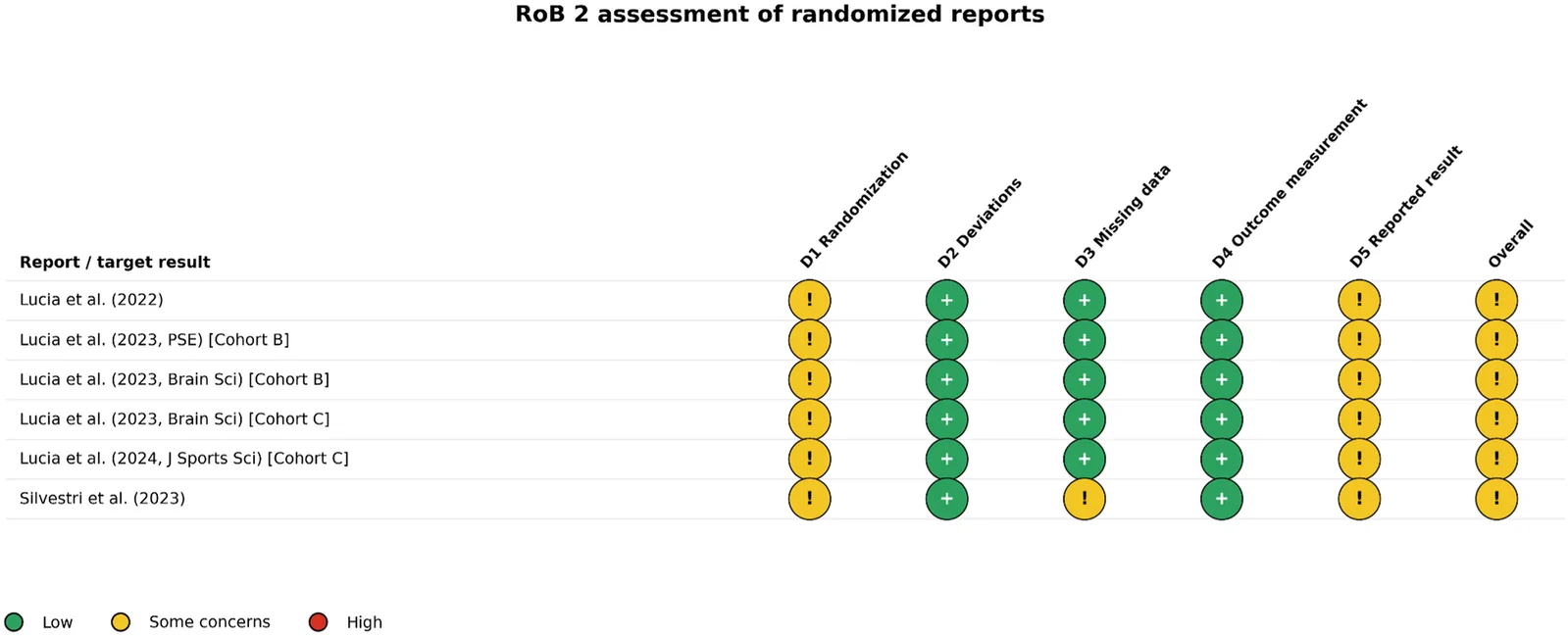
Traffic-light presentation of RoB 2 judgments for the randomized results.

**Figure 4:**
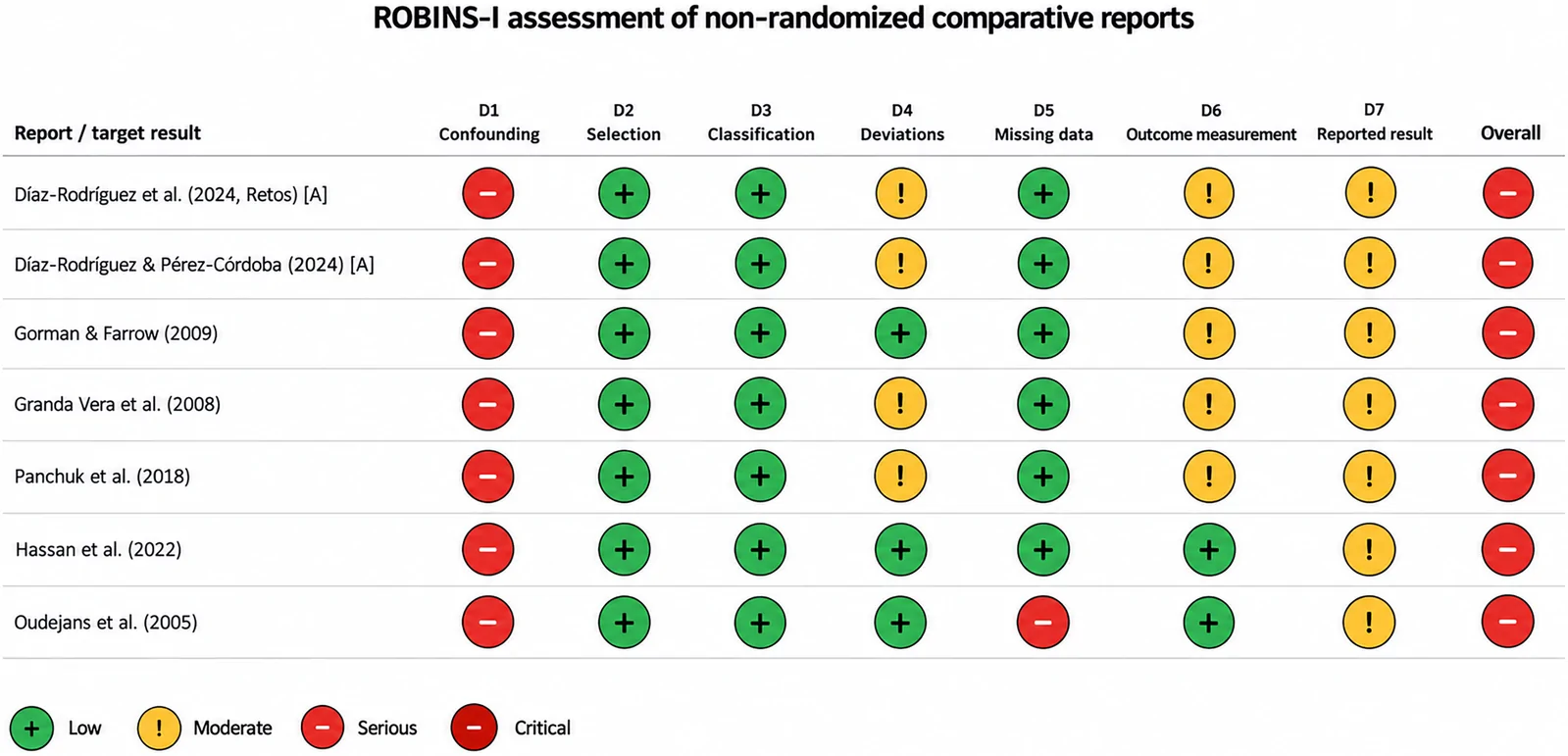
Traffic-light presentation of ROBINS-I judgments for the non-randomized results.

**Figure 5:**
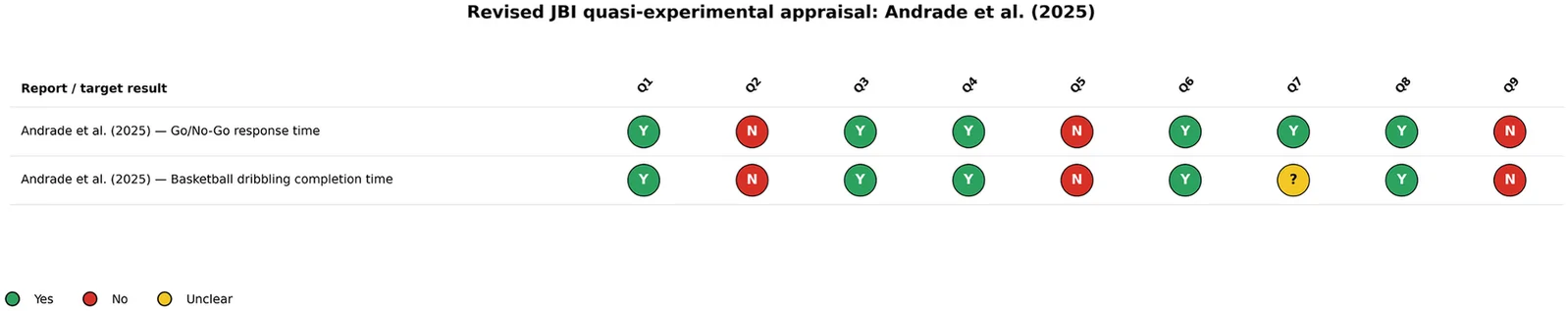
JBI quasi-experimental appraisal for the two selected outcomes.

For the randomized evidence, all six selected report-level results were judged to have some concerns overall, with RoB 2. The most recurrent uncertainty concerned the randomization process, particularly because the method used to generate or conceal the allocation sequence was incompletely described in several reports. Concerns regarding selection of the reported result were also common because studies frequently reported several eligible behavioral, performance, or neurophysiological outcomes and prospectively accessible analysis plans were generally unavailable. In contrast, there was little evidence that departures from the assigned intervention had materially biased the selected effects. Lack of participant or trainer blinding, which is often unavoidable in behavioral training studies, was therefore not treated as sufficient on its own to indicate bias due to deviations from intended interventions.

Missing outcome data were generally limited in the randomized reports. The main exception was Silvestri et al. (21), in which 58 players were randomized but nine were excluded during the intervention because of injury, COVID-19 vaccination, or illness, leaving 49 participants in the final analyses. This resulted in some concern regarding missing outcome data for the selected result. Outcome measurement itself was generally less problematic for objectively recorded behavioral or performance measures, although the availability of several possible outcome definitions and analyses continued to affect confidence in selective-result reporting.

The seven ROBINS-I assessments represented six unique non-randomized comparative cohorts. All six cohorts were judged to be at serious overall risk of bias for the selected intervention-effect result, with confounding remaining the principal limitation. Treatment allocation was linked to factors such as coaching decisions, team membership, participant availability, training commitments, or other non-random mechanisms that could also influence perceptual-cognitive or performance outcomes. For example, allocation in Gorman and Farrow (22) was constrained by transport and training commitments and subsequently involved quasi-random balancing according to playing position and baseline decision-making performance. Similarly, the Díaz-Rodríguez (13, 18) showed purposive allocation agreed with coaches rather than randomized assignment. Residual concerns were also identified for outcome measurement in studies relying on study-specific tactical judgments and for selection of reported results where several outcomes and analyses were available without a prospectively accessible analysis plan.

The uncontrolled study by Ximenes de Andrade et al. (23) was considered separately using the revised JBI quasi-experimental tool rather than being assigned a summary risk-of-bias score. The study preserved temporal ordering of intervention and outcome assessment and used the same participants and measurement procedures across phases; however, the six participants progressed through a fixed sequence of standard motor training, washout, and cognitive-motor dual-task training without a concurrent control group. There were also no repeated baseline and post-intervention measurement series around the cognitive-motor phase, and the analytical approach did not provide a strong counterfactual against which the specific effect of cognitive-motor training could be isolated. Accordingly, causal attribution of changes observed during the cognitive-motor phase remains limited.

### 3.4 Results of individual studies and synthesis

Table 3 summarizes the findings and the principal effect measures reported in each article. Findings were synthesized according to the primary perceptual-cognitive process targeted, while linked publications from the same cohort were considered together to avoid treating companion reports as independent evidence. For cue utilization and anticipation, findings were mixed. Granda Vera et al. (24) reported favorable changes in anticipation and defensive response performance, whereas Gorman and Farrow (22) found no clear advantage at post-test, retention, or competitive-game transfer. The Díaz-Rodríguez et al. (13, 18) showed improvements in tactical perception and tactical-solution quality following perceptual-tactical training, although the available analyses relied predominantly on within-group comparisons rather than direct between-group treatment effects.

**Table 3:** Summary of main results.

Perceptual-cognitive category	Study	Main findings	Effect measure
Cue utilization/anticipation	Granda Vera et al. (24)	The combined on-court cue instruction plus Reflex simulation condition produced the clearest pattern of benefit. At post-test, this group performed better than the control group on the anticipation ability index. The advantage remained at transfer, where both anticipation and defensive reaction time favored the simulation-enhanced condition.	Post-test, control vs. simulation + instruction: anticipation index, reported *U* = 2.16, *p* < 0.025; reaction time, not significant. Transfer: anticipation *U* = 2.31, *p* < 0.025; reaction time *U* = 2.78, *p* < 0.01. Experimental-B reaction time: 271.9 +/− 67.2 ms (pre), 259.5 +/− 68.9 ms (retest), 150.8 +/− 40.2 ms (post); within-group Friedman *χ*^2^ = 6.50, *p* < 0.025.
Cue utilization/anticipation	Gorman and Farrow (22)	Decision accuracy increased across the sample, but neither explicit nor implicit temporally occluded video training produced an intervention-specific advantage. The same pattern held at retention and during competitive-game transfer. The larger descriptive advantages in the two perceptual-training groups therefore remain suggestive rather than confirmatory.	Pre-post group x test: *F*(3,35) = 0.64, *p* = 0.60, *η*p^2^ = 0.05; time effect *F*(1,35) = 12.11, *p* = 0.001, ηp^2^ = 0.26. Descriptive gains: explicit +13.5%, implicit +13.4%, control +8.0%, placebo +4.14%. Retention interaction: *F*(3,34) = 0.64, *p* = 0.60, ηp^2^ = 0.05. Transfer interaction: *F*(3,25) = 1.16, *p* = 0.34, ηp^2^ = 0.12.
Cue utilization/anticipation	Diaz-Rodriguez et al. (18)	After 13 weeks, players receiving perceptual-tactical training identified more meaningful tactical information and gave fewer incorrect or incomplete solutions, while correct and original-positive solutions increased. The control group did not show comparable pre-post changes.	Tactical perception, experimental: 19.75 to 20.75 correct responses, *t* = −5.477, *p* < 0.01; control *p* = 0.08. Experimental tactical solutions: incorrect 3.56 to 2.69, *p* = 0.001; incomplete 2.81 to 2.13, *p* = 0.044; correct 18.00 to 18.93, *p* = 0.001; original-positive 0.63 to 1.13, *p* = 0.027. Corresponding control comparisons were non-significant.
Cue utilization/anticipation	Diaz-Rodriguez and Perez-Cordoba (13)	This companion report does not supply an independent training-effect estimate. Its added contribution is the analysis of individual differences in response to the same perceptual-tactical programme: stronger abstract reasoning was related to larger gains in tactical perception and to more original solutions, while psychoticism was associated with correct post-training tactical solutions.	Raven score predicting gain in accurate perceptions: *R* = 0.581, *F* = 7.134, *p* = 0.018. Correct tactical solutions: *R* = 0.358, *F* = 2.052, *p* = 0.174. Original solutions: *R* = 0.494, *F* = 4.508, *p* = 0.050. The study also gives *p* = 0.006 for Raven vs. original-positive post-test solutions and *p* = 0.036 for psychoticism vs. correct post-test solutions.
Decision-making/response selection	Panchuk et al. (6)	Immersive 360-degree decision training did not produce a clear overall advantage. Female players improved on the immersive test in both training and control groups, whereas the male training subgroup showed a large but non-significant gain. Small-sided-game results were similarly mixed: the omnibus interaction was significant, but follow-up contrasts did not isolate a treatment effect.	Immersive-test group x test x sex: *p* = 0.275. Female training: 38.3 +/− 3.1 to 45.7 +/− 4.9, *p* = 0.004, *d* = 1.79; female control *p* = 0.007, *d* = 2.71. Male training: 46.2 +/− 5.4 to 50.2 +/− 4.5, *p* = 0.127, *d* = 0.80. SSG interaction: *p* = 0.032; male training 20.2 +/− 9.3 to 26.1 +/− 6.3, *p* = 0.080, *d* = 0.74; female training *p* = 0.855, *d* = 0.06. Follow-up comparisons were non-significant.
Executive-control/cognitive-motor integration	Lucia et al. (25)	Basketball-specific cognitive-motor dual-task training produced clear intervention-specific gains in all five dribbling tests. Response consistency and commission errors also improved more in the CMDT group. Mean response time and omission errors changed over time but did not show a specific group-by-treatment effect.	Dribbling group x treatment interactions: ηp^2^ = 0.40 to 0.61, all *p* < 0.001 (crossover: *F*(1,22) = 26.4, ηp^2^ = 0.55). Response-time variability: *F*(1,22) = 8.6, *p* = 0.008, ηp^2^ = 0.28. Commission errors: *F*(1,22) = 7.0, *p* = 0.015, ηp^2^ = 0.24. Mean RT interaction *p* = 0.795, ηp^2^ < 0.01; omission-error interaction *p* = 0.110, ηp^2^ = 0.11.
Executive-control/cognitive-motor integration	Lucia et al. (8)	In the larger CMDT cohort, experimental players improved markedly on both pooled dribbling outcomes. Smaller, but still significant, intervention-specific effects were present for response time and commission errors. P3 amplitude also increased more after CMDT, whereas the earlier sensory components did not show the same intervention-specific pattern.	Single-change dribbling interaction: *F*(1,50) = 52.1, *p* < 0.001, ηp^2^ = 0.510; multiple-change interaction: *F*(1,50) = 71.2, *p* < 0.001, ηp^2^ = 0.588. RT interaction: *F*(1,50) = 6.44, *p* = 0.014, ηp^2^ = 0.114. Commission-error interaction: *F*(1,50) = 4.6, *p* = 0.036, ηp^2^ = 0.084. P3 in experimental group: 8.72 to 11.67 µV; post-test experimental vs. control *p* = 0.008.
Executive-control/cognitive-motor integration	Lucia, Aydin and Di Russo (19)	The sex-stratified companion analysis again found dribbling gains confined to CMDT. Some cognitive and preparatory brain responses differed by sex: response time improved most clearly in experimental females; false alarms decreased in experimental males and females, although control females also improved. The preparatory pN and BP responses likewise showed sex-dependent interactions.	Single-change group x test: *F*(1,48) = 55.2, *p* < 0.001, ηp^2^ = 0.535; multiple-change: *F*(1,48) = 82.1, *p* < 0.001, ηp^2^ = 0.631. RT group x test: *F* = 5.9, *p* = 0.018, ηp^2^ = 0.110; experimental females 458 +/− 63 to 415 +/− 53 ms, *p* < 0.001, ηp^2^ = 0.635. False-alarm group x test: F = 5.8, *p* = 0.020, ηp^2^ = 0.107. pN group x test: *F* = 24.9, *p* < 0.001, ηp^2^ = 0.341; BP group x test: *F* = 8.3, *p* = 0.005, ηp^2^ = 0.147.
Executive-control/cognitive-motor integration	Lucia et al. (20)	Adding cognitive tasks to the sprint-and-agility circuit produced intervention-specific improvements in 10-m sprint, change of direction, Go/No-Go response time and P3 amplitude. The matched motor-only programme showed little or no corresponding change over the five-week period.	Sprint interaction: *F*(1,28) = 16.0, *p* < 0.001, ηp^2^ = 0.363; experimental 2.54 +/− 0.10 to 2.41 +/− 0.11 s. Change of direction: *F*(1,28) = 11.4, *p* = 0.002, ηp^2^ = 0.289; experimental 5.92 +/− 0.34 to 5.73 +/− 0.28 s. RT: *F*(1,28) = 4.9, *p* = 0.035, ηp^2^ = 0.160; experimental 477 +/− 53 to 431 +/− 47 ms. P3 interaction: *F*(1,28) = 6.2, *p* = 0.021, ηp^2^ = 0.175; experimental 7.03 +/− 0.92 to 8.96 +/− 1.06 µV.
Executive-control/cognitive-motor integration	Lucia et al. (11)	This companion report reinforces the physical and response-speed findings from the same 30-player programme and adds a preparatory ERP analysis. Sprint, change-of-direction and response-time changes were reliable only in the physical-CMDT group. Motor preparation (BP) increased specifically after CMDT, whereas the pN and commission-error results did not show a clear intervention-specific effect.	Sprint group x intervention: *F*(1,28) = 33.66, *p* < 0.01, ηp^2^ = 0.54; experimental *d* = 0.65, SRD% = 3.34; post experimental vs. control *p* = 0.047, *d* = 0.23. Change of direction: *F* = 16.71, *p* < 0.01, ηp^2^ = 0.37; experimental *d* = 0.63, SRD% = 3.17. RT: *F* = 4.9, *p* = 0.03, ηp^2^ = 0.16; experimental *d* = 0.73, SRD% = 6.61. BP interaction: *F* = 7.92, *p* < 0.01, ηp^2^ = 0.22; experimental pre-post *p* = 0.012, *d* = 0.74. pN interaction *p* = 0.07; commission-error interaction *p* = 0.94.
Executive-control/cognitive-motor integration	Silvestri et al. (21)	Three weeks of massed basketball training improved several executive-function and fitness measures in both groups. Fitlight did not add a clear advantage for Flanker accuracy or response time. A small group-by-time signal appeared for forward Digit Span correct responses, and the Fitlight group also showed a stronger Yo-Yo IR1 trajectory and higher perceived session effort.	Flanker accuracy group x time: *F*(5.22,246) = 1.09, *p* = 0.369, ηp^2^ = 0.02; Flanker RT: *F*(8,376) = 1.12, *p* = 0.345, ηp^2^ = 0.02. Forward Digit Span correct-rate interaction: *F*(6.1,286) = 2.9, *p* = 0.008, ηp^2^ = 0.06. Yo-Yo IR1 interaction: *F*(1,47) = 5.09, *p* = 0.029, ηp^2^ = 0.10. Session-RPE interaction: *F* = 5.50, *p* < 0.001, ηp^2^ = 0.10.
Executive-control/cognitive-motor integration	Ximenes de Andrade et al. (23)	The sequential pilot did not demonstrate a statistically confirmed cognitive or sport-performance advantage for the dual-task phase. The largest changes were seen in two dribbling measures, but the design cannot separate CMDT effects from order, practice or carry-over effects.	Friedman/Kendall W across the four assessments: Double Crossover *χ*^2^ = 11.00, W = 0.611 (large); Between Legs + Behind the Back *χ*^2^ = 8.80, *W* = 0.489 (moderate). Go/No-Go RT W = 0.244 (small); accuracy *W* = 0.056 (negligible).
Attentional/gaze control	Oudejans et al. (27)	Visual-control training lengthened the period during which players picked up visual information before ball release and was accompanied by improved full-vision shooting. Competitive-game field-goal and three-point percentages increased across phases in the experimental players, whereas the comparison players did not show a corresponding systematic improvement. The evidence is nevertheless based on very small, outcome-specific samples, and no direct group-by-time treatment interaction was reported.	Final-period duration: 353 +/− 57.3 ms at the first laboratory session and 373 +/− 49.0 ms at the first 25 full-vision shots of the final session; overall condition effect *F*(4,16) = 9.98, *p* < 0.001, ES = 0.55; pre vs. post pairwise *t* = 3.32, *p* < 0.05, ES = 0.35. Full-vision shooting before vs. after goggle training: 57.2 +/− 7.1% vs. 62.8 +/− 11.3%, *t*(3) = 2.44, *p* < 0.05, ES = 0.78. Competitive field-goal percentage, experimental: 46.1 +/− 10.2% to 60.6 +/− 12.1%, *F*(2,6) = 5.30, *p* < 0.05, ES = 1.16; comparison: 42.5 +/− 3.7% to 42.2 +/− 3.6%, *F*(2,6) = 0.79, *p* > 0.10. Experimental three-point percentage: 35.2 +/− 6.6% to 53.9 +/− 7.8%, *F*(2,4) = 10.52, *p* < 0.05, ES = 2.75.
Decision-making/response selection	Hassan et al. (26)	Both groups improved over the eight-week period, but post-intervention reactive agility, bilateral visual reaction time and dribbling performance favored the FITLIGHT group. The analysis compared post-test group means with independent t-tests rather than estimating a group-by-time interaction, and no standardized between-group effect size was reported.	Post-test experimental vs. control (*n* = 10/group): agility, 10.18 +/− 0.08 vs. 10.83 +/− 0.15 s, *t* = 11.981, *p* < 0.001, difference in change ratio = 6%; visual reaction time-right, 0.307 +/− 0.01 vs. 0.370 +/− 0.48 (unit reported as ms), *t* = 3.079, *p* = 0.041, difference in change ratio = 14.1%; visual reaction time-left, 0.327 +/− 0.04 vs. 0.424 +/− 0.06 (unit reported as ms), *p* < 0.001, difference in change ratio = 14.1% (*t* = 4505); dribbling agility, 14.25 +/− 0.49 vs. 16.24 +/− 0.53 s, *t* = 8.775, *p* < 0.001, difference in change ratio = 11.7%. Experimental within-group changes were 11%, 23%, 31% and 19%, respectively.

Panchuk et al. (6) found no clear overall advantage of immersive video training for the primary decision-making outcome. Although some subgroup effects were sizeable, comparable changes in control participants and the absence of consistent follow-up contrasts limit causal interpretation. Representative small-sided-game transfer was similarly inconclusive.

The most consistent favorable signals occurred within executive-control and cognitive-motor integration interventions. Controlled cognitive-motor dual-task studies reported moderate-to-large interaction effects for several dribbling, sprint, change-of-direction, response-control, and selected neurophysiological outcomes. However, companion reports frequently arose from the same cohorts and therefore do not represent independent replications. Fitlight-enriched training showed limited additional benefit over basketball practice, while the uncontrolled Andrade et al. (23) provided only exploratory evidence because of its sequential design and small sample.

Attentional/gaze control was represented by one cohort. Oudejans et al. (27) reported changes in visual-information pickup during jump shooting together with improvements in laboratory and competitive shooting outcomes after visual-control training. However, analyses were based on very small outcome-specific samples, and a direct group-by-time treatment effect was not estimated, limiting causal interpretation.

Evidence for decision-making and response selection comprised two independent cohorts. Panchuk et al. (6) found no clear overall advantage of immersive video training for the primary decision-making outcome, and representative small-sided-game transfer was similarly inconclusive. Hassan et al. (26), by contrast, reported post-intervention differences favoring FITLIGHT training for visual reaction time, reactive agility, and dribbling performance. Both groups improved, however, and the study relied on post-test group comparisons rather than a group-by-time treatment estimate, so the magnitude of the intervention-specific effect remains uncertain.

Across categories, the evidence was strongest for trained-task performance and near-transfer outcomes. Retention, representative game-based transfer, and competitive-match transfer were rarely examined and did not show consistent intervention-specific benefits. Accordingly, the current evidence supports selected proximal performance effects more strongly than generalized improvements in basketball decision-making or competitive performance.

## 4 Discussion

This systematic review and evidence gap map provide an overview of perceptual-cognitive training in youth basketball players. Three patterns are most important. First, the most consistent favorable signals came from executive-control and cognitive-motor interventions, particularly for dribbling, sprint, change-of-direction, and selected response-control outcomes. Second, cue-utilization and anticipation interventions produced mixed findings across studies and testing contexts. Third, direct evidence for decision-making training was sparse and did not show a clear overall intervention effect. Across all categories, the literature was concentrated on performance measured in the trained task or in closely related laboratory and drill-based tests. Retention, representative game-based transfer, and competitive-match transfer were uncommon and did not show a consistent intervention-specific benefit. The main contribution of the review is therefore not evidence that perceptual-cognitive training works as a single intervention class, but a clearer description of which processes have been trained, where favorable effects have been observed, and where the evidence becomes thin.

Video, immersive displays, interactive lights, and other devices can impose quite different perceptual-cognitive demands, and the same device can be used for tasks with different learning objectives. The synthesis therefore separates the perceptual-cognitive target from the delivery modality. This distinction also helps explain why results cannot be interpreted simply by comparing technologies. The available studies are better understood in terms of the information that players must detect or select, the response they must generate or inhibit, and the extent to which these requirements are coupled to basketball action.

### 4.1 Attentional/gaze control, cue utilization, anticipation, and decision-making: proximal learning versus transfer

The cue-utilization and anticipation evidence illustrates why a transfer hierarchy is necessary. Granda Vera et al. (24) reported favorable changes after combining on-court advance-cue instruction with computer simulation, including better anticipation and defensive response performance on a more demanding assessment. In contrast, Gorman and Farrow (22) found larger descriptive improvements after explicit and implicit video training but no intervention-specific advantage at post-test, after the retention interval, or during competitive-game assessment. The two Díaz-Rodríguez reports (13, 18) described improvements in tactical perception and tactical-solution quality after perceptual-tactical training, but the principal intervention relied mainly on within-group comparisons rather than a direct group-by-time treatment estimate (18), while the companion analysis focused on individual characteristics associated with response to the same programme rather than providing an independent intervention effect (13). These studies cannot therefore be treated as convergent replications of a single effect.

The decision-making evidence was even more limited. Panchuk et al. (6) used immersive 360-degree video with elite youth players and included an on-court small-sided-game assessment, but the primary decision-making analysis did not show a clear overall training advantage. Some subgroup changes were large, yet comparable changes in control players and non-significant follow-up contrasts made the treatment effect difficult to isolate. This matters because improvement on the training display, or on a task that closely resembles it, is not equivalent to improved decision-making in game play. The present findings are broadly compatible with the wider team-sport literature, where perceptual-cognitive interventions tend to show larger effects on laboratory or task-specific outcomes than on real-game performance (15). The current basketball evidence narrows that observation further since the problem is not simply whether a training method produces change, but how far that change travels from the practiced information-action relation.

Representative learning design offers a useful way to think about this issue, but the present review does not establish representativeness as a moderator of effectiveness. A task should not be considered representative merely because it looks like basketball or uses basketball footage. More relevant questions concern whether the information variables that normally regulate action are available, whether realistic action possibilities are preserved, and whether perception remains coupled to an executable response. These features were not formally scored across the included studies, so any suggestion that more representative tasks transfer better should remain a hypothesis rather than a review finding. It is also possible that more representative tests are less controlled and therefore noisier, particularly when opponents, teammates, and tactical context vary between assessments. The null competitive-transfer finding of Gorman and Farrow (22), for example, may reflect limited transfer, limited measurement sensitivity, or both. Future work will need to separate these possibilities rather than assuming that ecological appearance alone guarantees valid transfer measurement.

### 4.2 Executive-control and cognitive-motor integration

The most consistent favorable pattern in the review came from interventions that integrated a defined cognitive demand with basketball-relevant movement. The most consistent favorable pattern in the review came from interventions that integrated a defined cognitive demand with basketball-relevant movement. Across the Lucia studies (8, 11, 19, 20, 25), cognitive-motor dual-task training was associated with moderate-to-large interaction effects for several dribbling, sprint, and change-of-direction outcomes, together with intervention-specific changes in selected response-control and neurophysiological measures. Importantly, these reports did not represent five independent replications. Reports (8, 19) arose from the same 52-player cohort, reports (11, 20) arose from the same 30-player cohort, and report (25) represented a separate cohort. The companion publications therefore provide complementary characterization of the same intervention responses rather than independent confirmation of the observed effects.

Several Lucia papers were companion reports from the same participant cohorts (8, 20, 25), so they provide richer characterization of the same intervention response rather than independent replication. In addition, most favorable outcomes were measured in dribbling, sprint, change-of-direction, or discrimination-response tasks. They are therefore best regarded as trained-task or near-transfer effects. None of the executive-control/cognitive-motor cohorts contributed representative game-based or competitive-match transfer evidence in the revised map. The current evidence supports adaptation under closely related cognitive-motor demands more clearly than it supports generalized improvement in game decision-making.

The less favorable studies are informative in this respect. Silvestri et al. (21) found that a short period of massed basketball training improved several outcomes in both groups, while Fitlight enrichment produced little additional benefit for the principal executive-function measures. Similarly, the uncontrolled sequential pilot by Ximenes de Andrade et al. (23) did not establish a cognitive or sport-performance advantage attributable specifically to the dual-task phase, and the fixed intervention order leaves practice and carry-over effects unresolved. These findings challenge the notion of a simple dose-of-cognitive-load explanation. Adding stimuli or a secondary task is unlikely to be sufficient on its own. The cognitive demand must be identifiable, meaningfully linked to the motor task, and compared against an active condition that controls for basketball practice and training exposure.

The neurophysiological findings also require careful wording. Changes in P3, Bereitschaftspotential, prefrontal negativity, or related components occurred alongside behavioral and motor changes in some reports (8, 20, 25), but co-occurrence does not establish mediation. Demonstrating that a neural or attentional change explains a performance effect would require a temporal model in which the proposed mediator is measured at appropriate points, followed by an adequately powered mediation or other causal-process analysis. The present studies provide mechanistic signals and useful targets for future work, but they do not show that neurophysiological adaptation caused the observed basketball-performance changes.

### 4.3 Evidence distribution and developmental relevance

The 14 reports represented 11 unique participant cohorts once companion publications were accounted for. Five cohorts primarily examined executive-control or cognitive-motor integration, three focused on cue utilization or anticipation, two on decision-making or response selection, and one on attentional/gaze control. The five executive-control/cognitive-motor cohorts all contributed general cognitive or behavioral and near-transfer outcomes, with three also reporting neurophysiological measures, yet none assessed representative game-based or competitive-match transfer. Across the entire map, formal retention remained represented by only one cohort, representative game-based transfer by one cohort, and competitive-match transfer by two cohorts.

There was a predominance of male samples, while the mixed-sex evidence came largely from a small number of studies. The same applies to playing level and geography since several reports were derived from a single high-level Italian basketball environment, while the remaining evidence came from Spain, Australia, and Brazil. Analyses focusing on differences between sexes are of interest, yet they remain based on one cohort and should not be read as evidence of stable sex differences in training responsiveness (19).

### 4.4 Methodological limitations and confidence in the findings

All randomized results were judged as having some concerns with RoB 2, most often because the randomization process was incompletely described and because the choice among multiple available outcomes or analyses was not always supported by an accessible prospective analysis plan. Importantly, inability to blind participants or trainers was not treated as a source of bias by itself. The non-randomized comparative cohorts were all judged to be at serious overall risk of bias in ROBINS-I, with confounding the predominant limitation. Allocation in some studies was influenced by coach decisions, team membership, participant availability, or training commitments, all of which can also be related to baseline performance or subsequent improvement. The uncontrolled Andrade study (23) was considered through item-level JBI appraisal and, because it lacked a concurrent control and used a fixed intervention sequence, offers only weak counterfactual support for attributing change to the cognitive-motor phase.

These judgments affect the interpretation of the intervention patterns. Some favorable cue-utilization and tactical findings came from non-randomized studies in which residual confounding cannot be separated cleanly from the intervention effect. Conversely, the strongest cognitive-motor signals were observed in randomized comparisons, but several reports came from overlapping cohorts. A pooled effect estimate was not considered defensible for the present evidence base. Within cue-utilization and anticipation, the interventions ranged from on-court cue instruction combined with simulation to temporal-occlusion video and perceptual-tactical slide-based training; comparators, outcome definitions, and analytical approaches also differed, and direct between-group estimates were not consistently available. The decision-making/response-selection category contained only two independent cohorts, with substantial differences in intervention modality, outcome measurement, and analytical approach. Within executive-control/cognitive-motor integration, several reports were linked to the same samples and the outcomes spanned dribbling, sprint, change of direction, Go/No-Go behavior, working memory, and event-related potentials. Pooling these measures into a common effect would imply a level of construct equivalence and independence that the studies do not support. For these reasons, the structured synthesis emphasizes effect magnitude where reported, comparative design, transfer level, and risk of bias rather than producing a summary estimate that would be difficult to interpret.

### 4.5 Practical implications and priorities for future research

Coaches considering cognitive-motor dual-task work have the strongest current evidence for proximal outcomes such as dribbling, sprint, change of direction, and response control when the cognitive demand is embedded within basketball-relevant movement. These interventions may be useful as adjuncts to technical and physical preparation, but the evidence does not show that they improve overall game intelligence or competitive decision-making. Similarly, video, simulation, or immersive methods may be useful for cue recognition and decision practice, yet the present youth evidence is too limited and inconsistent to claim a reliable advantage over conventional training. The device itself should not be treated as the active element.

The next generation of studies should be designed around explicit learning and transfer questions. A minimum outcome structure would distinguish performance on the trained task, retention after a meaningful delay, near transfer to an untrained or modified task, representative game-based transfer, and competitive-match transfer. This hierarchy should be specified before data collection, with the criterion task chosen to reflect the intended practical claim. When representative transfer is the objective, investigators should describe the information variables available to the player, the action possibilities preserved, the fidelity of the required response, and the extent of opponent or teammate interaction. Such reporting would allow representative learning design to be tested rather than invoked retrospectively.

Methodological priorities are equally clear. Independent, adequately powered randomized trials are needed, preferably across more than one team or academy, with transparent sequence generation and allocation procedures, prospective analysis plans, adherence and attrition reporting, and effect estimates accompanied by confidence intervals. Head-to-head studies could be particularly informative if they manipulate delivery modality while holding the perceptual-cognitive target constant; for example, comparing two-dimensional and immersive decision training using equivalent scenarios would help separate the effect of technology from the effect of the trained information. Dose-response work is also needed because the present interventions varied substantially in session duration, frequency, and total exposure.

Mechanism studies should go beyond collecting cognitive and neurophysiological outcomes alongside performance measures. If attentional control, inhibition, or preparatory neural activity is proposed as a mediator, repeated measurements must establish temporal ordering and the analysis must test whether change in the proposed mediator accounts for subsequent performance change. The same rigor is needed for developmental questions. Studies should characterize biological maturation, training age, playing experience, sex, and competitive level *a priori* and be powered for interaction analyses when moderation is claimed. Broader representation of female players, different development pathways, and additional geographical contexts would improve external validity. These steps would move the field from demonstrating task-specific responsiveness toward explaining when perceptual-cognitive training produces durable and behaviorally meaningful change.

## 5 Conclusions

The evidence base supports proximal benefits of basketball-specific perceptual-cognitive training in pediatrics and youth players, with the clearest favorable signals arising from executive-control and cognitive-motor interventions targeting closely related movement and response-control outcomes. Cue-utilization and anticipation findings were mixed, and direct decision-making evidence was sparse. Retention, representative game-based transfer, and competitive-match transfer remain major gaps, so the current literature does not establish generalized improvement in basketball decision-making or durable competitive performance. Confidence is further limited by small samples, overlapping reports, heterogeneous outcomes, and risk-of-bias concerns, particularly in non-randomized studies. Future progress depends on independent randomized trials that distinguish acquisition from progressively farther transfer, report effects transparently, and test developmental and mechanistic hypotheses with designs capable of supporting those inferences. From the point of view of sports sciences and pediatrics, this review clarifies the current evidence base for perceptual-cognitive training in youth basketball and highlights the need for developmentally sensitive trials that account for age, sex, training experience, and competitive level when evaluating learning and transfer.

## Data Availability

The original contributions presented in the study are included in the article/Supplementary Material, further inquiries can be directed to the corresponding author.
